# Recent Advances in the Applications of Water‐soluble Resorcinarene‐based Deep Cavitands

**DOI:** 10.1002/open.202200026

**Published:** 2022-06-14

**Authors:** Yu‐Jie Zhu, Ming‐Kai Zhao, Julius Rebek, Yang Yu

**Affiliations:** ^1^ Supramolecular Chemistry & Catalysis and Department of Chemistry College of Science Shanghai University Shanghai 200444 China

**Keywords:** confinement, deep cavitand, molecular container, molecular recognition, synthetic host

## Abstract

We review here the use of container molecules known as cavitands for performing organic reactions in water. Central to these endeavors are binding forces found in water, and among the strongest of these is the hydrophobic effect. We describe how the hydrophobic effect can be used to drive organic molecule guests into the confined space of cavitand hosts. Other forces participating in guest binding include cation−π interactions, chalcogen bonding and even hydrogen bonding to water involved in the host structure. The reactions of guests take advantage of their contortions in the limited space of the cavitands which enhance macrocyclic and site‐selective processes. The cavitands are applied to the removal of organic pollutants from water and to the separation of isomeric guests. Progress is described on maneuvering the containers from stoichiometric participation to roles as catalysts.

## Graphical abstract

**Calix[4]resorcinarene‐based water‐soluble deep cavitands** and their applications in molecular recognition, isomers separation, sensing and removal of organic pollutants and reaction vessels are summarized and discussed.

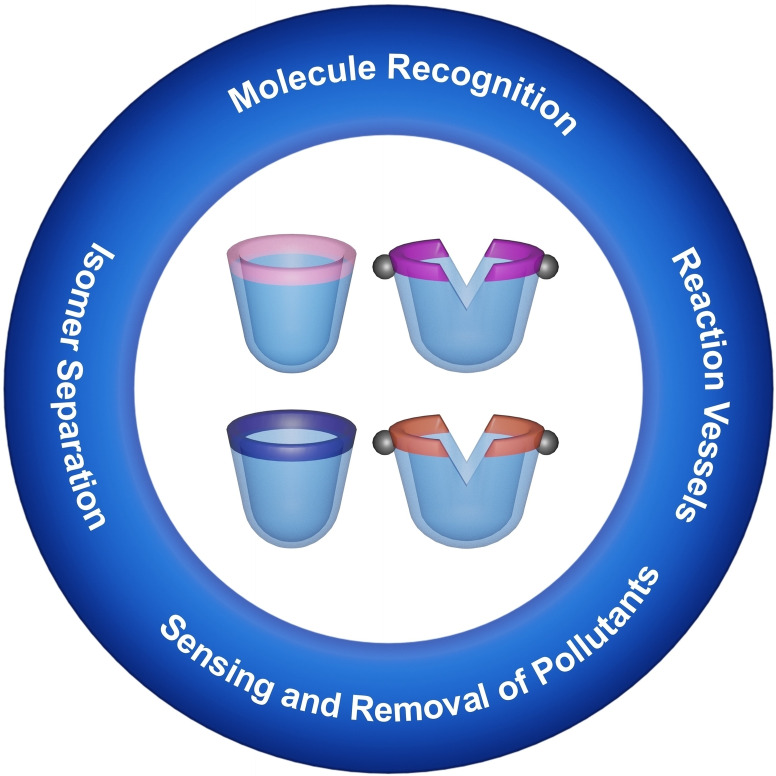

## 1 Introduction

Heightened awareness of the environmental problems presented by the continued consumption of fossil fuels has stimulated the search for alternatives.^[1]^ In organic chemistry, much of the use is in solvents and nothing is more attractive and more obvious than replacing organic reaction media with water.^[2]^ But problems with accessible acids and bases, diminished polar binding forces and general insolubility of many organic compounds in water make direct substitution of solvent with water unworkable. Moreover, many catalysts “drown” in water, one reason why enzymes hide their catalytic sites in “dry” environments in Nature.^[3]^ Synthetic container molecules such as capsules provide a haven for small guests by providing a physical barrier between the cavities inside and the solvent outside.^[4]^ The containers come with built‐in hydrophobic interiors, as a result of the aromatic panels used for their construction. While convenient for synthetic purposes, aromatics provide little in the way of functional groups that can bear directly on the guest inside.^[5]^ This is an unintended consequence of the encapsulation process. Cavitands, by way of their open ends, provide access of reagents in solution to exposed parts of their guests,^[6]^ and water‐soluble cavitands direct which parts of their guests are exposed.^[7]^ Earlier, we described how this works for macrocyle synthesis chaperoned by cavitands,^[8]^ here we develop these notions to subtle applications in guest recognition and reaction selectivity.

## 2 Binding Selectivity in Water‐soluble Cavitands

### 2.1 Orientations of α, ω‐Difunctional Alkanes

**Figure 1:**
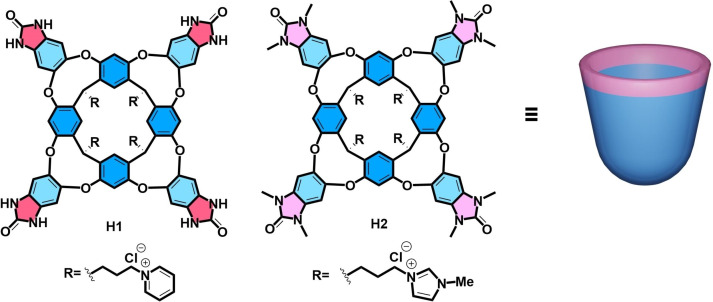
Chemical structures of cavitands **H1** and **H2**, and the cartoon used for cavitand **H2**.

Water‐soluble synthetic deep cavitands, tetra‐urea cavitand **H1** ^[9]^ and its exhaustively methylated version, cavitand **H2**, unable to dimerize into a capsule (Figure 1),^[10]^ have been extensively studied in molecular recognition and as confined spaces for reactions.^[11]^ These two deep cavitands possess a highly preorganized, nonpolar hydrophobic binding pocket, lined with π‐faces of eight electron‐rich aromatic rings. In this regard, they resemble the active sites of enzymes, offering a range of chemical environments, from hydrophilic to hydrophobic. Guest molecules with complementary size, shape and chemical surface can be included in the confined space, and amphiphilic guests assume a predictably biased arrangement. Accordingly, even subtle differences in the polarity of isomeric guests can be sensed in their host−guest complexes through NMR spectroscopy.

**Figure 2:**
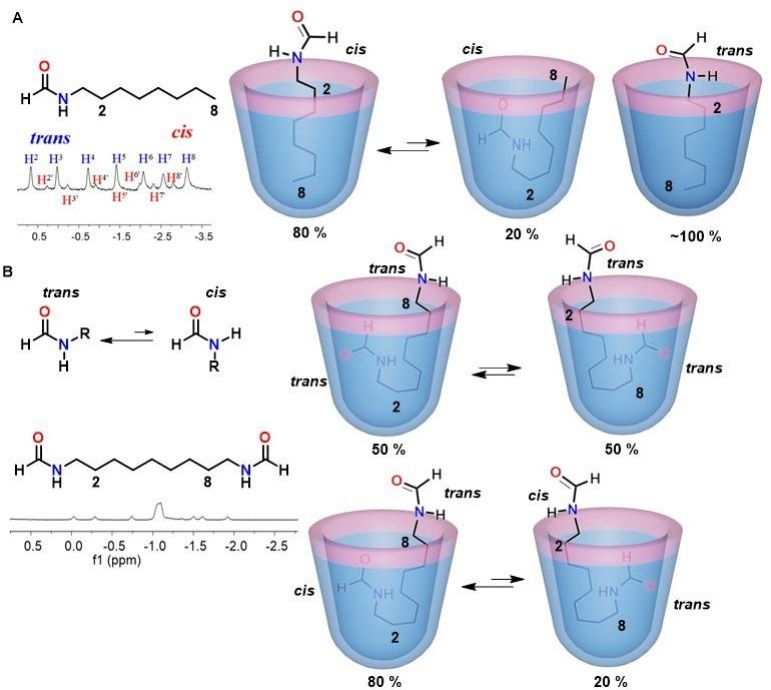
A: Partial ^1^H NMR spectrum for a monformamide and cartoons of its behavior in cavitand **H2**. B: Partial ^1^H NMR spectrum for a diformamide and cartoons of its behavior in cavitand **H2**.

Formamides exist as a mixture of both *cis* and *trans* isomers and cannot be separated under ordinary conditions owing to their rapid interconversion on the human timescale.^[12]^ Recently, we determined the relative hydrophilic differences between isomeric formamides by binding in the synthetic cavitand **H2** through direct competition experiments.[^13^, ^14^] Long‐chain mono‐ and bis‐formamides were sequestered in the deep cavitand, driven by hydrophobic forces. The complexes show two sets of NMR signals in the upfield regions corresponding to the *trans*‐ and *cis*‐isomers (Figure 2). However, the caviplexes of diformamides showed starkly different signal patterns to that of the monoformamides. The major *trans*,*trans*‐bis‐formamide isomers presented simplified spectra indicating a time‐averaged symmetrical arrangement in the cavity, and the minor *trans*,*cis*‐bis‐formamide isomers presented a superimposed set of signals featuring an unsymmetrical arrangement, in which one end spends more time inside the cavitand than the other (Figure 2B). The biased arrangement of the unsymmetrical *trans*,*cis* guest arises from the differences in relative hydrophilicity and hydrophobicity of the two ends. According to the NMR spectroscopic assignments of both mono‐ and bis‐formamides, the terminal *trans*‐formamides spend more time outside the cavitand than the *cis*‐isomers, revealing that *cis*‐formamides are more hydrophobic than the corresponding *trans*‐ counterparts.

The cation−π interaction^[15]^ and the hydrophobic eﬀect^[16]^ are important intermolecular forces for complex formation in water and play major roles in controlling recognition properties in biological systems. One question that arises in given situations concerns which driving force is stronger; the cation−π or the hydrophobic eﬀect. The cation−π interaction is attractive in the gas phase and nonpolar organic solvents;^[17]^ nevertheless, the situation in water is more complicated. Molecular recognition in water involves competition with solvent interactions and the particularly significant desolvation.^[18]^ Many biomolecular complexation experiments suggest that, in water, the complex involving the ammonium⋅aromatic interaction is more stable than that featuring the *t*‐butyl⋅aromatic interaction; however, most often the charged residues of biomolecules can compromise the experimental results. Synthetic host systems inspired by natural receptors provide an opportunity to elucidate the fundamental properties of noncovalent interactions in a stripped‐down context that removes the complexity associated with the functional group diversity and conformational dynamics existing in biomolecules.

**Figure 3:**
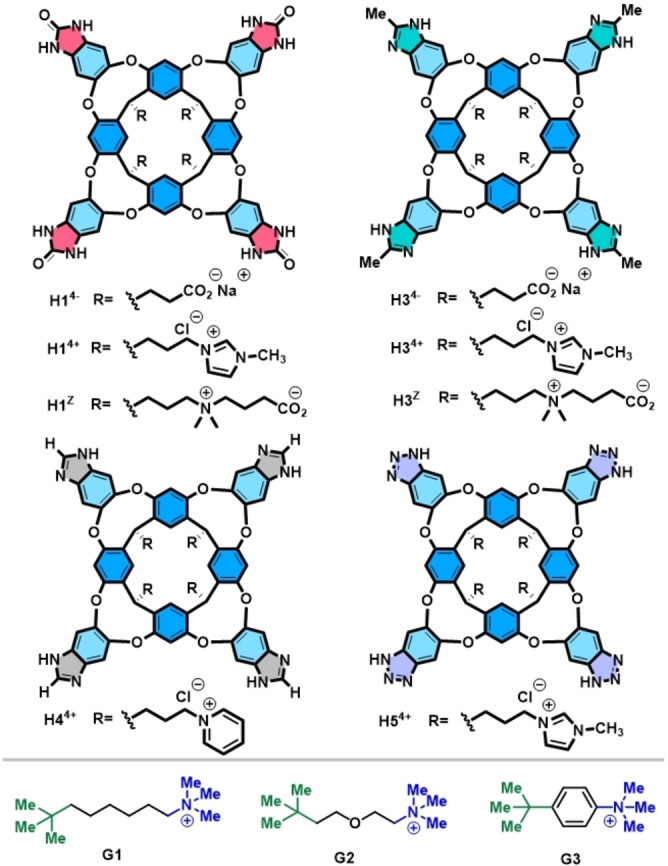
Chemical structures of eight cavitand hosts **H1**, **H3**, **H4**, **H5** and three symmetric dumbbell guests **G1‐**‐**G3**.

**Figure 4:**
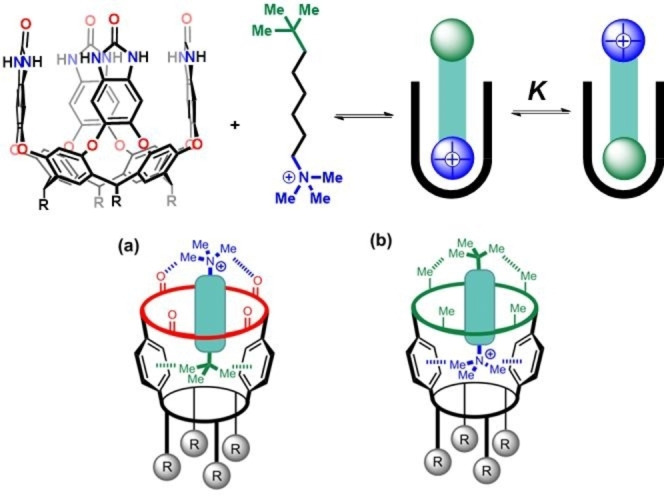
Top: Schematic representation of the two isomeric complexes formed between cavitand hosts and dumbbell guest **G1**, K is defined as the equilibrium constant in favor of the t‐butyl‐in isomer. Bottom: For guests with short linkers, the external head group comes into contact with the upper rim of the cavitand hosts. (a) The polar urea groups in **H1** (red) make attractive interactions with the cationic head group. (b) The hydrophobic methyl groups in **H3** (green) attractively interact with the *t*‐butyl head group. Partially reproduced with permission from Ref. [19], Copyright 2021, American Chemical Society.

Very recently, we synthesized eight different deep cavitand hosts with the same binding pocket but diﬀering in the polarity of the functional groups that line the entrance to the binding site and bearing different charges on the peripheral water‐solubilizing groups (Figure 3). Direct intramolecular competition experiments using dumbbell guests – having a trimethylammonium group at one end and a *tert*‐butyl group at the other – were devised to measure the relative contributions of cation−π interactions and hydrophobic effects to the complexation (Figure 4).^[19]^ Unexpectedly, the container molecules consistently preferred binding to the uncharged *tert*‐butyl group of the dumbbell guest with the longest linker **G1**. This occurred regardless of the charge on the host or the polarity of the functional groups that line the entrance to the binding pocket. This overwhelming preference is determined by the solvation of the polar trimethylammonium group in water, which outcompetes the attraction between the positive charge and the *π*‐surfaces in the container. The cavitand complexes provide a direct measure of the relative strengths of cation−π interactions and desolvation in water, in which interactions with the uncharged *tert*‐butyl group are more than 12 kJ mol^−1^ more favorable than the cation−π interactions with the trimethylammonium group.

Shorter dumbbell guests **G2** and **G3** showed more complicated behavior. The ^1^H NMR spectra of their complexes with cavitands **H1**, **H4**, and **H5** indicate that the *t*‐butyl end of the guest is preferentially bound in the cavitand pocket, and the trimethylammonium group is exposed to water, resembling those shown for **G1**. In contrast, the complexation with cavitand **H3**, there are two sets of signals corresponding to the *t*‐butyl end bound and the trimethylammonium end bound: both of the isomeric complexes are present. Molecular modeling was used to provide some insight into the diﬀerence in binding behavior observed for these complexes. It revealed that when the length of the linker is shorter, the attractive interactions between polar trimethylammonium group and the rim of cavitands **H1** increases the stability of this isomer. The hydrophobic interactions between the nonpolar *t*‐butyl group and the rim of the cavitands **H3** favor the cation−π isomer (Figure 4). These additional interactions between the cavitand and the head groups perturb the equilibrium between the two isomeric complexes.

### 2.2 Binding Selectivity with Aromatics and Others

**Figure 5:**
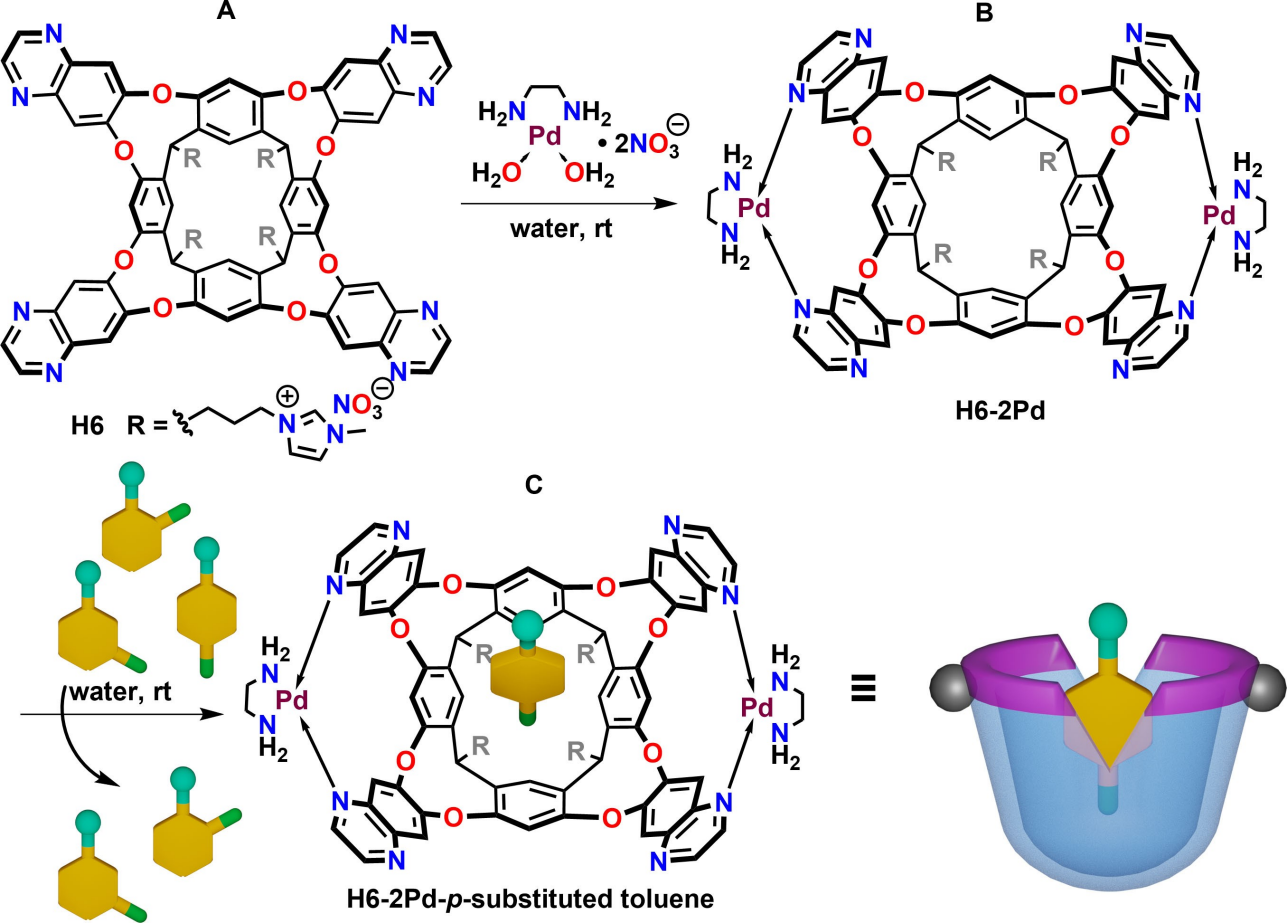
Structures of cavitand **H6** (A) and its dimetallic palladium(II) complex **H6**−**2Pd** (B) and representation of generic selective binding of *p*‐functionalized toluenes in competition with *o*‐ and *m*‐isomers (C).

The energy‐efficient separation of alkylaromatic compounds is a major industrial sustainability challenge, and separations of *p*‐xylene from its *o*‐ and *m*‐isomers was classified as one of the “seven chemical separations to change the world”.^[20]^ During the past decade, many energy‐efficient adsorption materials have been developed to meet the separation challenge, including zeolites,^[21]^ carbon molecular sieves,^[22]^ metal‐organic frameworks (MOFs),^[23]^ and solid‐state pillar[n]arenes.^[24]^ Nevertheless, approaches based on molecular recognition are scarce, and there seems to be no example of liquid−liquid phase separation for *p*‐xylene and related positional isomers. Recently, we reported a water‐soluble cavitand **H6** ^[25]^ and its palladium(II) derivative **H6**−**2Pd** (Figure 5).^[26]^ The coordination rigidifies the cavity and stabilizes the receptive vase shape,[^27^, ^28^] which imparts a welcome selectivity in guest binding. We applied the restricted volume of metallo‐cavitand **H6**−**2Pd** and its dynamics to separate *p*‐functionalized toluenes from their isomers in liquid–liquid extraction processes (Figure 5).^[29]^ All the functionalized toluene *p*‐isomers showed the methyl group was bound deep in the cavity with a 1 : 1 stoichiometry of the host and guest. Competitive binding experiments indicate that the selectivity was exclusive and the *p*‐ isomer can be separated efficiently from a mixture using cavitand **H6**−**2Pd**.

**Figure 6:**
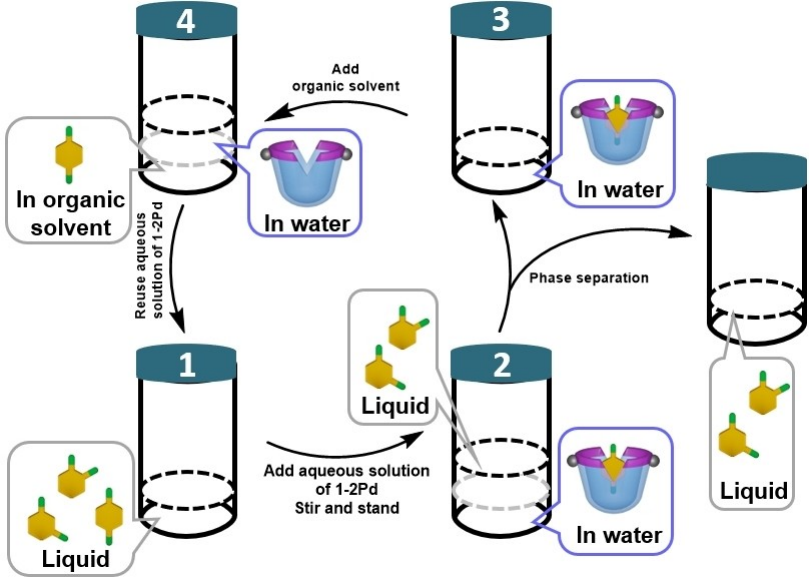
General liquid−liquid separation scheme for *p*‐xylene.

Based on the binding selectivity, liquid−liquid extraction experiments^[30]^ were carried out for the separation of a typical mixture of xylene isomers obtained from crude oil distillation (1 : 3 : 1 for *o*‐:*m*‐:*p*‐isomers) (Figure 6), and also *p*‐nitrotoluene from its isomers. The binding and liquid–liquid extraction of the xylene mixture was quantified and the recyclability of this metal cavitand system was also confirmed.

**Figure 7:**
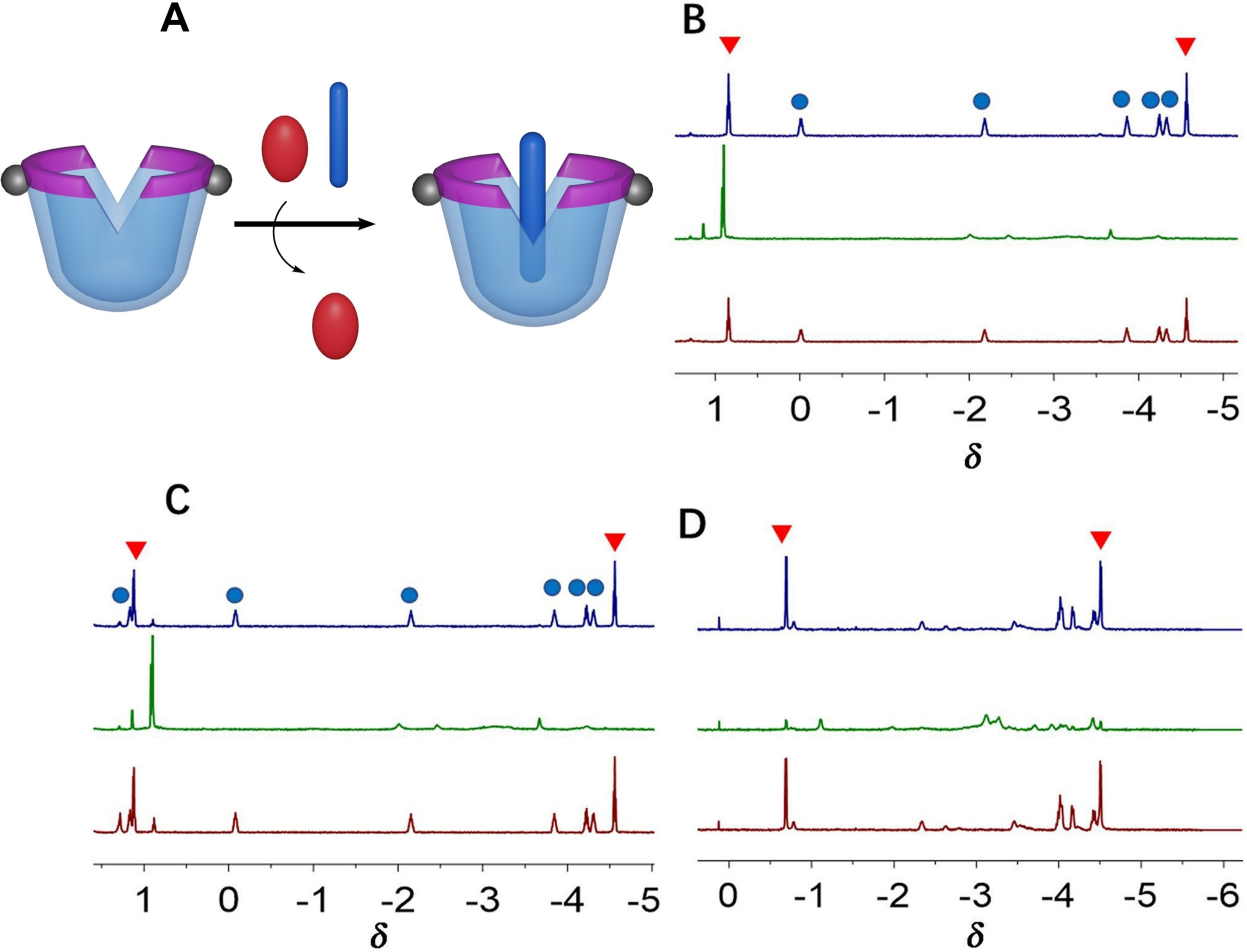
Cartoons (A) and partial comparative ^1^H NMR spectra (B, C, D) for cavitand **H6**−**2Pd** selectively binding an *n*‐alkane in competition with isooctane (chemical shifts are given in ppm). The blue rod represents straight alkanes, the red oval represents isooctane. Methyl protons are marked with red triangles and methylene protons are marked with blue dots. Partially reproduced with permission from Ref. [31], Copyright 2021, Wiley.

Alkanes are also important industrial feedstocks obtained from fossil fuels and traditionally separated by energy‐intensive fractional distillation. Very recently, we showed that metallo‐cavitand **H6**−**2Pd** can also be applied to the selective sensing and separation of straight and branched chain alkanes in energy‐efficient processes, which involves gas−liquid adsorption and liquid−liquid extraction.^[31]^ Competitive binding experiments and NMR spectroscopy showed the overwhelming preference of this synthetic water‐soluble cavitand host for the hydrophobic guests that possess narrow or flat shapes (Figure 7).^[31]^ For additional separation applications, interphase adsorption and liquid−liquid phase extraction were performed, and the alkane mixtures were efficiently separated. In addition, cavitand **H6**−**2Pd** can also be used as a recyclable synthetic cavitand host for selective separation of *n*‐alkanes from isooctane in an energy‐efficient manner.

**Figure 8:**
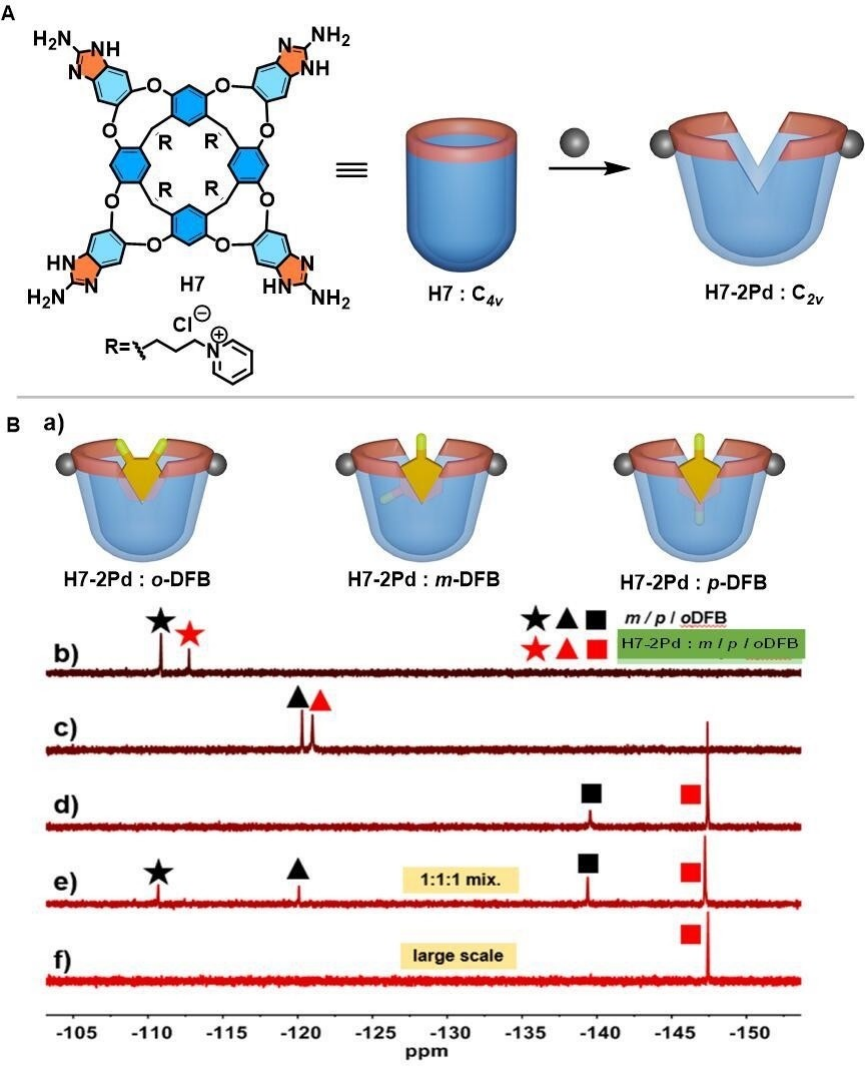
A: Structure and cartoons of the cavitands **H7** and its palladium complex **H7−2Pd**. B: (a) Cartoons of proposed models of binding of the difluorobenzene regioisomers to metallo‐cavitand **H7−2Pd**. Comparative partial ^19^F NMR spectra of the complexes formed between host **H7−2Pd** and DFB regioisomers: (b) bound *m‐*DFB, (c) bound *p‐*DFB, (d) bound *o‐*DFB, (e) excess 1 : 1 : 1 DFB mixture and (f) cavitand **H7−2Pd**. Partially reproduced with permission from Ref. [33], Copyright 2021, American Chemical Society.

Many structural variants of cavitands have been reported that are based on the resorcinarene platform, with additional functional groups on the upper rim for further possibilities in selective guest recognition.^[32]^ We recently expanded the applications of metal complexation by using 2‐aminobenzimidazoles for the walls of the cavitand (Figure 8A).^[33]^ The reorganized shape of the space in metallo‐cavitand **H7−2Pd** showed a good shape and size selectivity for *o*‐difluorobenzene over its isomers. Competitive experiments concerning the binding of difluorobenzene isomers to cavitand **H7−2Pd** used ^19^F NMR spectroscopy and revealed their stabilities decreased in the following order: *o*‐difluorobenzene>*p*‐difluorobenzene ≫ *m*‐difluorobenzene (Figures 8B, 8 C). Furthermore, based on the recognition selectivity, *o*‐difluorobenzene was also effectively separated from its regioisomers in liquid–liquid extraction process. The aqueous solution of cavitand **H7−2Pd** was reused to perform another extraction cycle.

**Figure 9:**
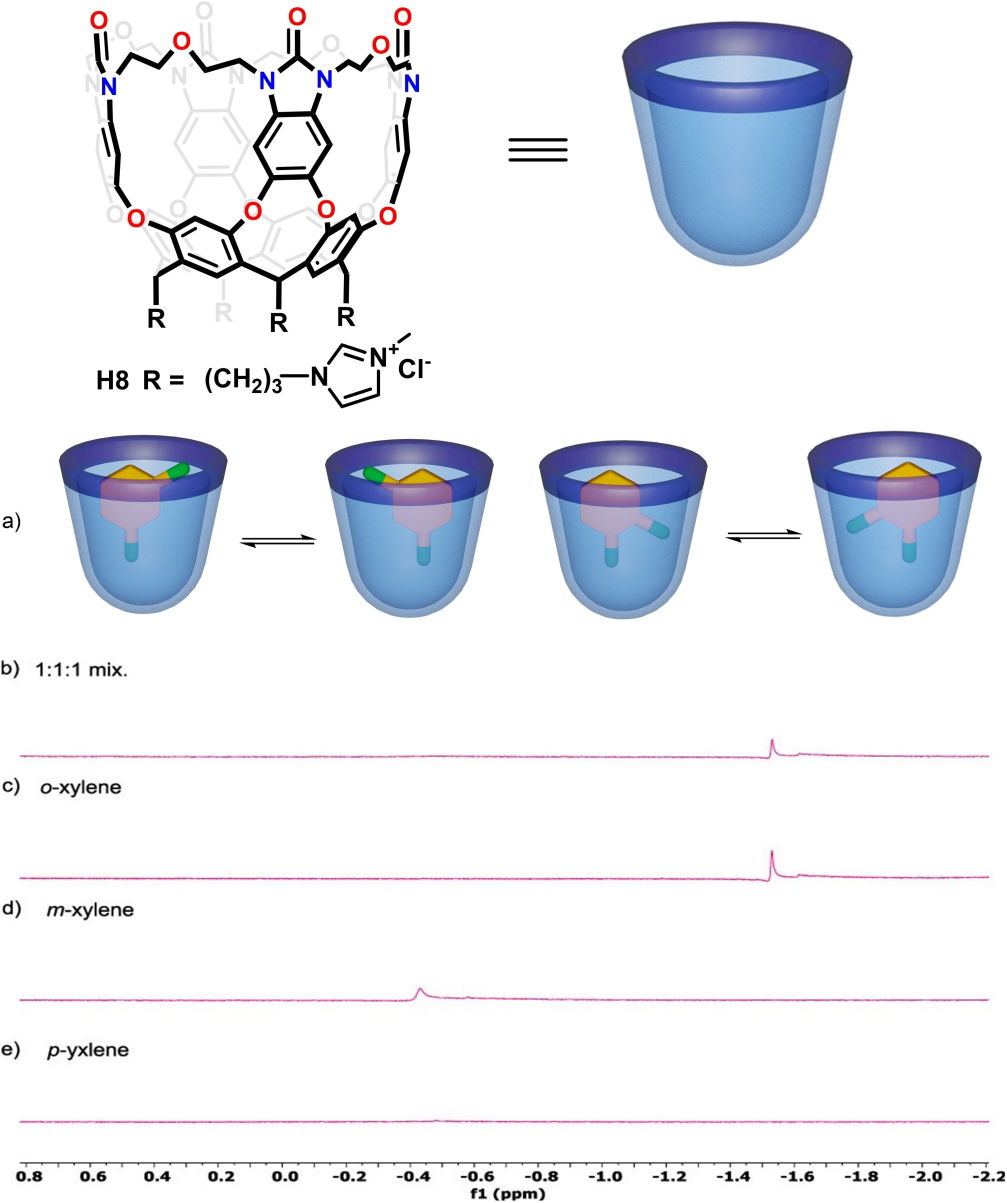
Top: Chemical structure and cartoon of cavitand **H8**. Bottom: (a) Cartoons of proposed binding models for the *o*/*m*‐xylene to cavitand **H8**. Comparative partial ^1^H NMR spectra of the complexes formed between host **H8** and excess xylene isomers: (b) mixture (1 : 1 : 1) of xylene isomers; (c) *o*‐xylene; (d) *m*‐xylene; (e) attempt with *p*‐xylene. Partially reproduced with permission from Ref. [35], Copyright 2021, American Chemical Society.

It has been generally accepted that cavitands with more rigidified walls in their receptive vase shapes often show slower dynamics of guest exchange and a better selectivity in guest binding. Such is the case with Gibb's deep cavitand having covalent and rigid walls that pre‐organize its shape.^[34]^ To control the shape and size of the container's cavity and suppress the kite conformers, a new water‐soluble dynamic cavitand **H8** with covalently bridged benzimidazolones at the upper rim was prepared (Figure 9).^[35]^

In contrast to dynamic cavitands **H1** and **H2**, the flexible spacers of cavitand **H8** cannot fold to the unreceptive kite shape. The motions of the walls are somewhat reduced, resulting in altered recognition properties. In D_2_O, cavitand **H8** can form stoichiometric complexes with any *o*‐ and *m*‐xylene isomers, and competitive binding experiments showed the stability order was *o*‐xylene>*m*‐xylene. Surprisingly, the *p*‐xylene isomer showed no binding (Figure 9), which is in contrast to the narrower metallo‐cavitand **H6**−**2Pd** that shows high selectivity for this isomer. Based on the unexpected selectivity for the *o*‐xylene isomer, cavitand **H8** was applied to the separation of a typical mixture of xylene isomers obtained from crude oil distillation with a liquid‐liquid extraction process.

**Figure 10:**
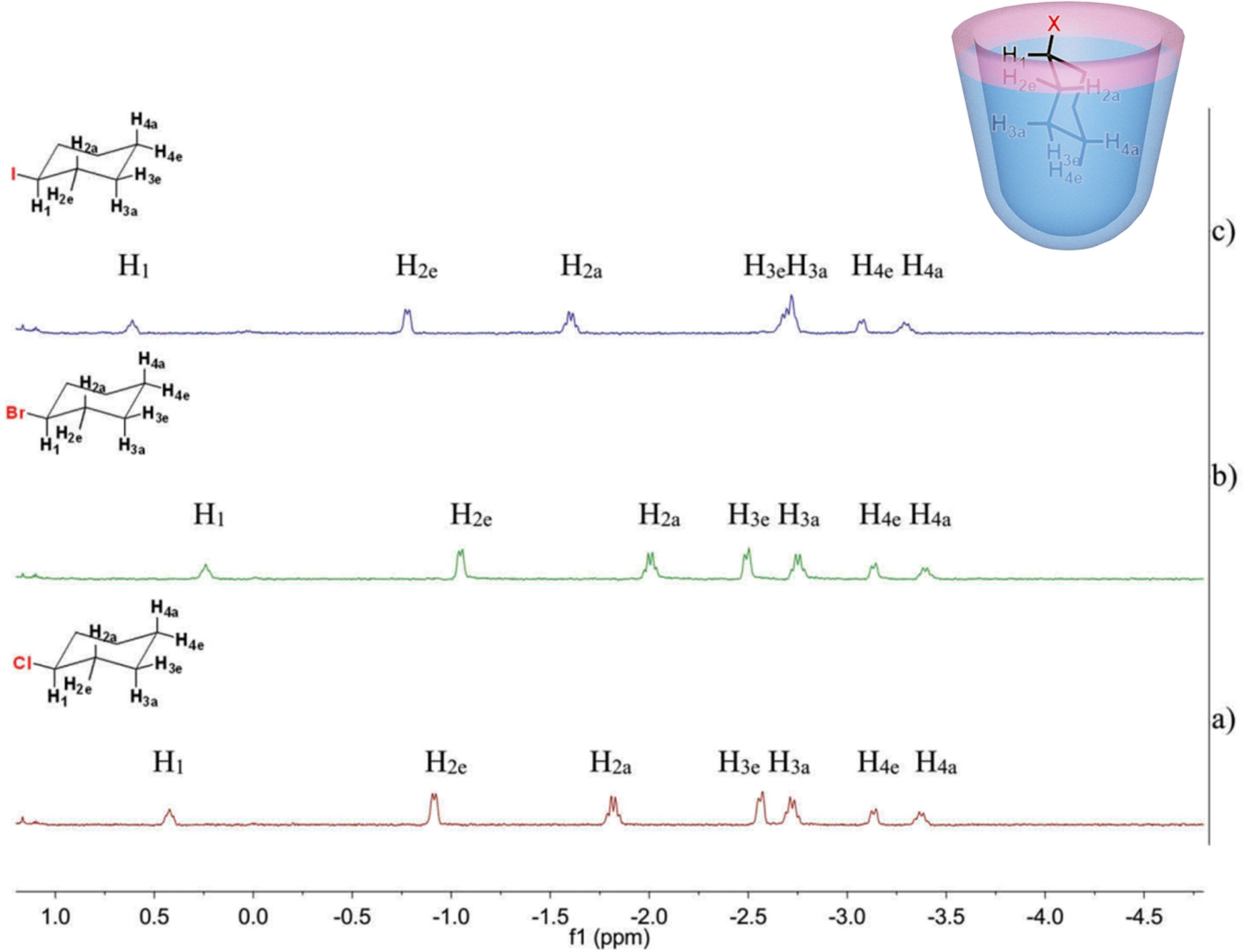
Left: Partial ^1^H NMR spectra of host‐guest complexes with **H2** (600 MHz, 298 K, D_2_O) and (a) 1‐chlorocyclo‐hexane; (b) 1‐bromocyclohexane; (c) 1‐iodocyclohexane. Right: Cartoon for cavitand **H2** and cyclohexyl halide complexes. Partially reproduced with permission from Ref. [37], Copyright 2019, Taylor&Francis.

In recent years, a greater awareness concerning the hazardous nature of halogen‐containing organic compounds and their roles in several human and animal diseases has grown.^[36]^ Meanwhile, interest in reducing toxic chemicals released into aqueous environments by sequestration is also growing. We used octamethyl‐urea cavitand **H2** as a sensor for monitoring cycloalkyl halides and 2‐methylisoborneol, a common drinking water pollutant.[^37^, ^38^] Cycloalkyl halides with diﬀerent halogen atoms (chloro, bromo or iodo) can qualitatively form the corresponding host−guest complexes with cavitand **H2**, which significantly reduces their characteristic smells. The presence of halogen atoms leads to preferred orientations in the cavity and completely diﬀerent ^1^H NMR spectra of the host−guest complexes (Figure 10).

Moreover, the rotation of the cyclohexyl halides is also slowed due to the steric hindrance of the halogen atoms. 2‐Methylisoborneol^[39]^ was also captured quantitatively by cavitand **H2** at concentrations lower than 1 mm, a behavior that suggests the potential of cavitand **H2** in water purification.

**Figure 11:**
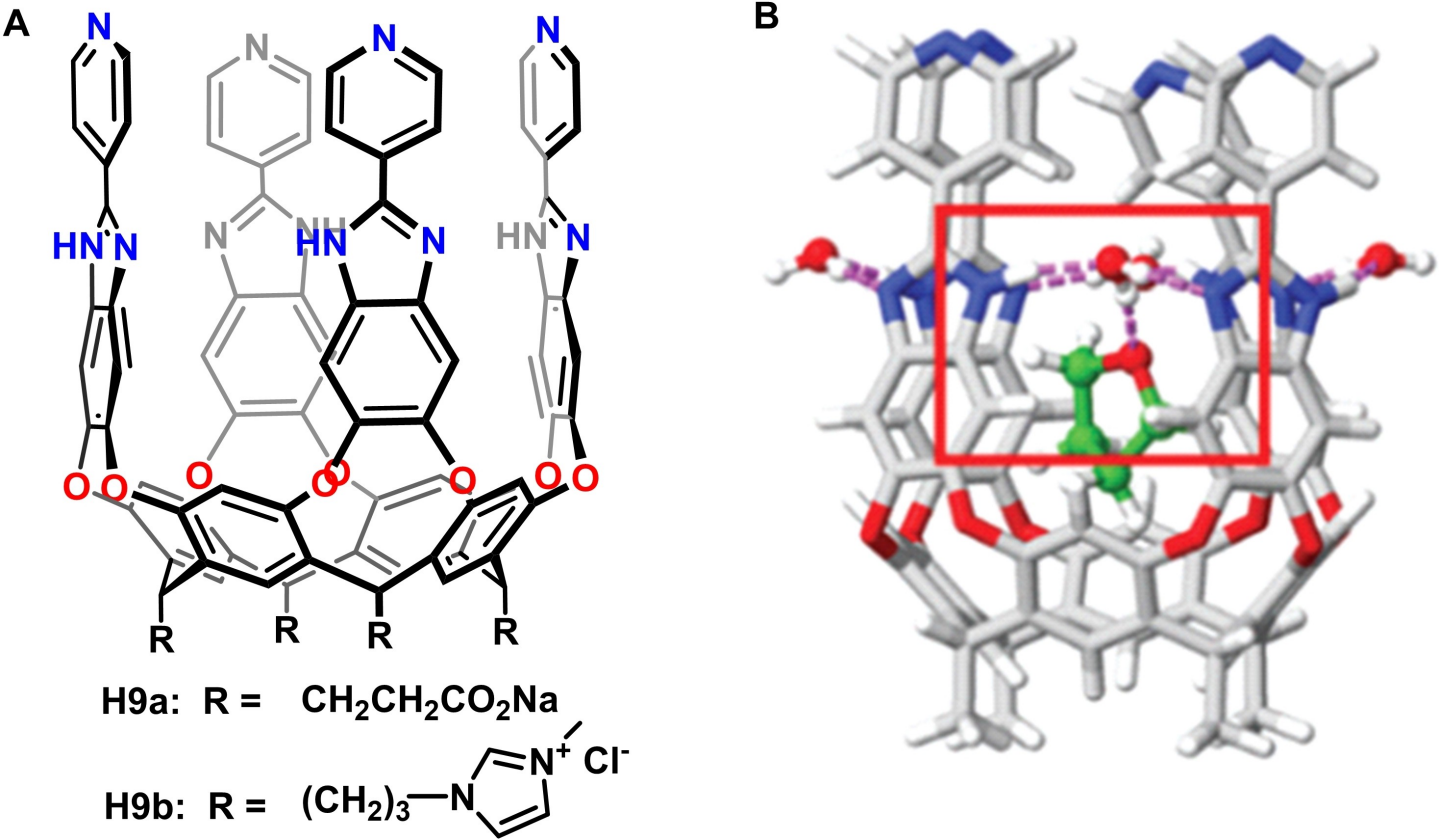
A: Chemical structures of cavitands **H9**. B: Water‐mediated hydrogen bonding interactions between host−guest complex. Partially reproduced with permission from Ref. [42], Copyright 2021, Royal Society of Chemistry.

Selective recognition of hydrophilic molecules in water is also a challenge for synthetic molecular receptors,^[40]^ and few supramolecular structures are up to this task.^[41]^ However, hydrophilic biomolecules, such as sugars, can be recognized by natural receptors, even with high selectivity, through cooperative action of hydrophobic eﬀects and other non‐covalent interactions, such as hydrogen bonds. There are few, if any, synthetic supramolecular systems that mimic this interaction, and very recently, we undertook the task to incorporate water‐mediated hydrogen bonds for molecular recognition in host−guest chemistry.^[42]^ New 2‐pyridylbenzimidazole‐functionalized water‐soluble cavitands **H9** possessing more extended aromatic walls and deeper hydrophobic spaces were synthesized (Figure 11A). The high aﬃnity, water‐mediated hydrogen bonds enhance the molecular recognition of neutral hydrophilic molecules, such as 1,3‐dioxane, THP, oxepane, THF and 1,4‐dioxane. All of these host−guest complexes generally show slow rates of in/out guest exchange on the NMR time scale.

In addition, the signals in the upfield region suggest that the oxygen atoms of bound heterocycles prefer positions away from the walls and near the polar parts of the cavitand. Isothermal titration calorimetry (ITC) experiments showed high association constants (*K*_a_) – up to 6.32×10^4^ m ^−1^ for the complexation of THF and 1.09×10^4^ m ^−1^ for 1,4‐dioxane. Quantum chemical calculations were also employed to optimize the host−guest geometries and showed that, in all cases, no hydrogen bonds could be formed directly between the guest and the NH groups of the benzimidazole. Instead, the oxygen atoms of the guests were engaged in one or two hydrogen bonds, with either water molecules at the seam (i. e., those bridging the benzimidazole panels) or water molecules hydrogen‐bonded to them (Figure 11B). Accordingly, the synergistic action between hydrogen bonding and hydrophobic eﬀects accounts for the high aﬃnity and selectivity.

**Figure 12:**
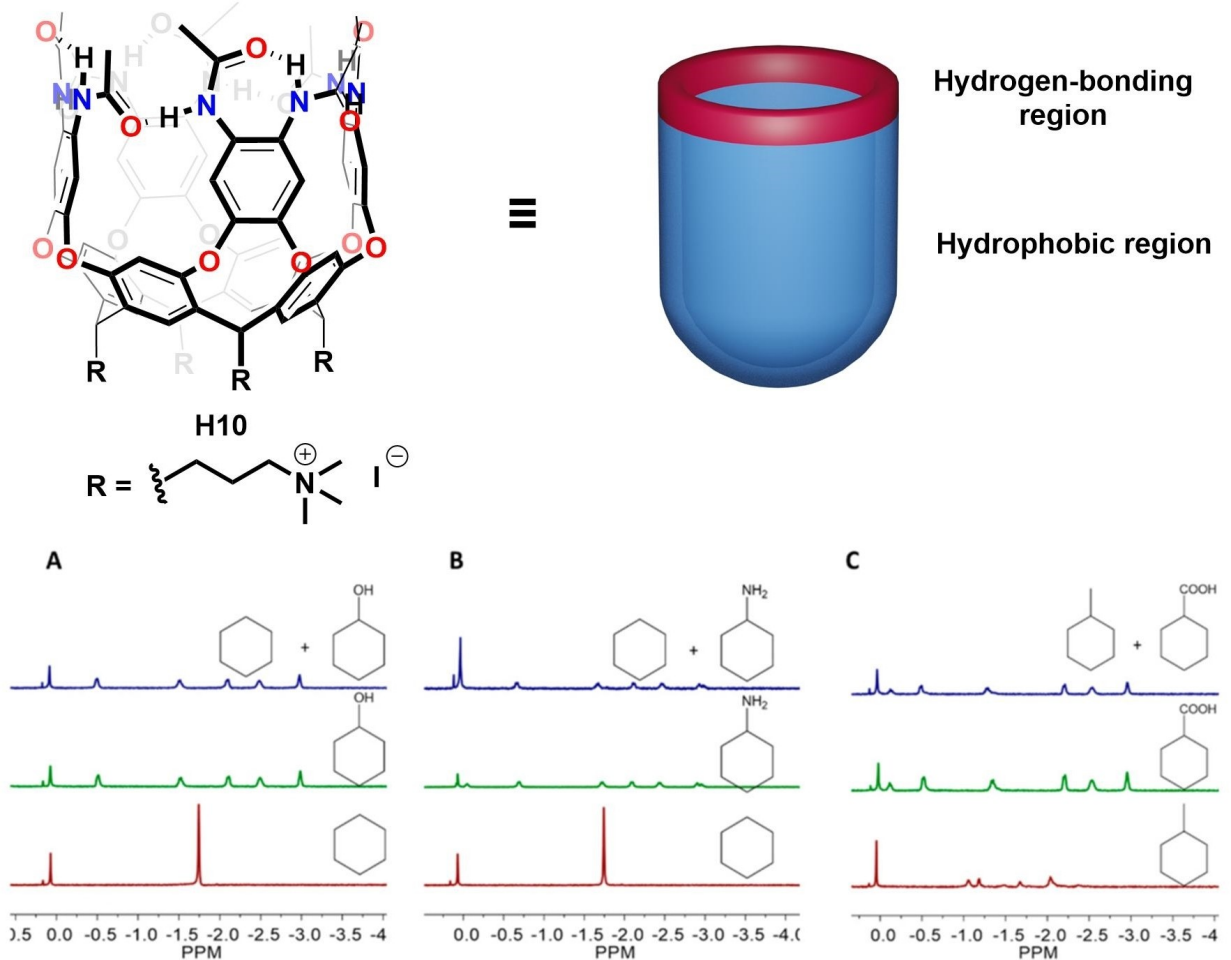
Top: Chemical structures and the cartoon depiction of cavitand **H10**. Bottom: Partial comparative ^1^H NMR spectra of cavitand **H10** upon adding from bottom to top cyclohexane, hydrophilic cyclic guests, and excess 1 : 1 mixture of cyclohexane and its hydrophilic analogue.

Similar work on the selective recognition of hydrophilic cyclic compounds, such as cyclohexyl alcohols, amines and acids in water by a new cavitand **H10**, has also been reported (Figure 12).^[43]^ The cavitand bears octa‐amides on the upper rim and trimethylammonium groups on the feet. Interestingly, competitive binding experiments indicate that cavitand **H10** shows higher binding affinity to hydrophilic cyclic molecules, compared to their hydrophobic counterparts of similar sizes and shapes (Figure 12). To some extent, this contradicts the usual experience that the hydrophobic effect is the main driving force attracting the guest into the cavity. The explanations for the selectivity can be attributed to the synergistic action of hydrogen bonding and the hydrophobic effect. Cavitand **H10** has both a hydrogen bonding region on the upper rim and a hydrophobic region composed of four aromatic walls and the resorcinarene core. When molecules with both nonpolar parts and polar functions are encountered, cavitand **H10** provides a hydrophobic environment to accommodate the hydrophobic surface of the guests, and the amides on the upper rim supply hydrogen bond donors and acceptors to complement the guests’ polar functional groups.

## 3 Reactivity in Water‐soluble Deep Cavitands

In bulk solution, identical functional groups at the end of long chain substrates tend to behave independently: reactivity at one site is not aﬀected by the remote site, showing essentially statistical reactivity and resulting in low selectivity. Therefore, the monofunctionalization of identical functional groups far away from each other is a true challenge and many efforts have been made to obtain selective processes for symmetric substrates. Encapsulation of a suitable guest in confined spaces forces orientations inaccessible in solution and can lead to unexpected reaction selectivity for the guest.^[44]^ Water‐soluble synthetic host molecules oﬀer a range of environments to their guests: polar functions of guests are exposed to the medium, while hydrophobic groups are generally buried deep in the containers and hidden from reagents in solution. We have reported the use of water‐soluble cavitand **H2** as reaction vessels and blocking groups.

**Figure 13:**
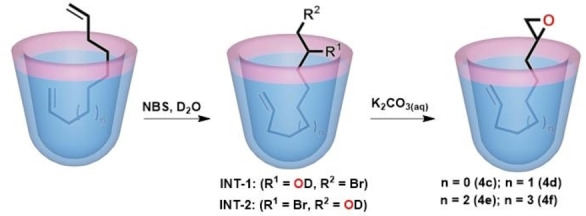
Cartoons of the epoxidation process with NBS (*N*‐bromosuccinimide) and base with cavitand **H2** in aqueous medium.

Mono‐functionalizations, such as the hydrolysis of long‐chain diesters,^[45]^ synthesis of macrocyclic ureas,^[46]^ the Staudinger reactions of diazides^[47]^ and the monohydrolysis of long chain α,ω‐dibromides have been reported earlier.^[48]^ Recently, we again performed the mono epoxidation of α,ω‐dienes in the presence cavitand **H2**, and excellent selectivity was obtained (Figure 13).^[49]^ Reaction of one end of a diene with NBS in water gives a bromohydrin intermediate that binds in the cavitand with the hydroxyl exposed and the remaining alkene buried assuming a J‐shape orientation in the cavity. Treatment with base converts the bromohydrin to an epoxide, completing the mono‐epoxidation process. With linear aliphatic dienes, the yield reached up to 84 %, and for 1,4‐diisopropenyl benzene, a nearly quantitative yield of the monoepoxide was obtained. The application should be general for converting long‐chain, symmetrical hydrophobic guests to unsymmetrical, amphiphilic ones.

Free radicals are highly reactive species with fast kinetics and low discrimination. For such species, the selectivity of product distribution is usually a challenge, and only a few selective radical processes have been reported in supramolecular hosts.

**Figure 14:**
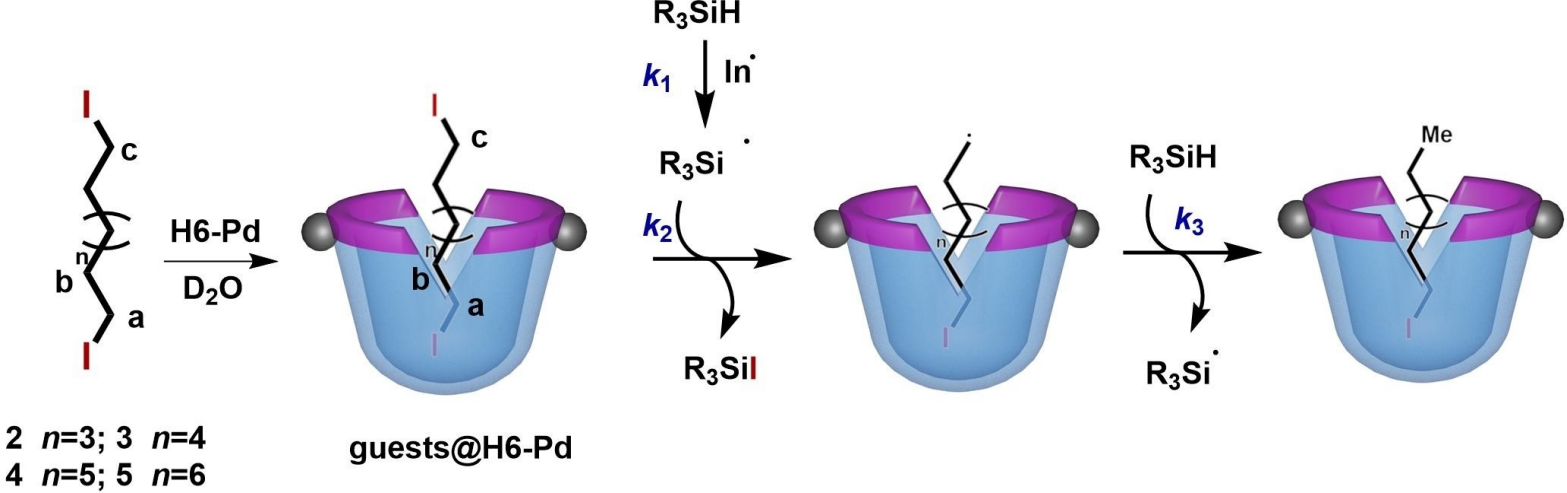
General scheme for the radical reduction of bound guests in cavitand **H6**−**2Pd** using a general reducing agent (R_3_SiH).

The Ramamurthy group investigated radicals generated in capsular hosts,[^50^, ^51^] and showed that confinement plays an important role on product selectivity.^[52]^ Our recent studies revealed the effects of guest affinity on radical processes in dynamic cavitand **H2** and metallo‐cavitand **H6**−**2Pd**.^[53]^ Here, in order to give further information of the effects of confinement on fast radical reactions (k≥10^3^ m^−1^ s^−1^), the reduction of alkyl dihalide guests with trialkylsilanes (R_3_SiH) was performed in a water‐soluble metallo‐cavitand **H6**−**2Pd** (Figure 14).^[54]^ Excellent conversion (>94 %) and high selectivity (>95 %) for the mono‐reduced products were obtained for both primary and secondary dihalides, even when 2 or 3 equivalents of the reducing agents were used. In bulk solution, only low selectivity could be observed. These results highlight the role of confinement for product selectivity in radical processes.

**Figure 15:**
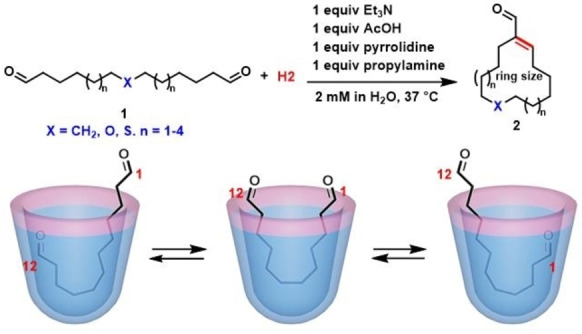
Up: selective intramolecular aldol condensations in cavitand **H2**. Bottom: Cartoons for dialdehydes@**H2**.

End‐to‐end intramolecular cyclization of long‐chain linear precursors is generally hard to achieve and often unpredictable because of unfavorable entropic problems and competitive intermolecular reactions. Beyond the mono‐functionalizations described above, template effects are also featured in complexes where intramolecular reactions can occur through control of guest conformation.[^55^, ^56^, ^57^] Several reactions including macrolactonization chaperoned by the cavitand **H2** have been reported,[^44^, ^58^, ^59^] including a recent application in the intramolecular aldol/dehydration reaction of long‐chain, linear dialdehydes in aqueous solution (Figure 15).^[60]^ Symmetric long chain α,ω‐dialdehydes are bound in host cavitand **H2** by hydrophobic forces. The NMR spectra show folded, J‐shape conformations with “yo‐yo motions” that favor macrocyclization reactions over intermolecular reactions observed in bulk solution. Linear dialdehydes, including heteroatomic dialdehydes, reacted smoothly, and 11‐ to 17‐membered macrocyclic products were isolated in good yields (30–85 %) and good selectivity. Unlike conventional templates with convex structures that become guests inside their assembled hosts, cavitands with concave surfaces reverse the roles and resemble the situation in biological catalysis – the templates are hosts for guests undergoing the assisted reaction processes.

## 4 Conclusions

In summary, this mini‐review focuses on the most recent advances of resorcin[4]arene‐derived water‐soluble deep cavitands. It includes their applications in molecular recognition, isomeric guest separations, sensing and removal of organic pollutants and their use as reaction vessels. Molecular recognition is the basis for distinguishing the subtle differences between isomers, determining the relative strength of cation−π and hydrophobic eﬀects in water, sequestering small hydrophilic molecules and separating complex mixtures. Some fundamental principles of noncovalent interactions were also determined from the study of these synthetic host‐guest systems, which enhanced our understanding of both chemical and biological molecular recognition phenomena. Guests confined in the limited spaces of synthetic containers can adopt unusual orientations, which leads to unexpected reactivity and changes in their chemical and physical properties. Mono‐functionalization and macrocyclization were successfully reported, in which water‐soluble cavitand hosts act as protecting groups, templates and reaction vessels. While these examples constitute encouraging applications of synthetic molecular containers, some problems still need more attention. Future cavitand research should address applications of these molecules as catalysts rather than as recyclable stoichiometric agents. Additionally, the development of resorcin[4]arene‐based cavitands as molecular sensors outside laboratory settings remains a challenge.

## Conflict of interest

The authors declare no conflict of interest.

5

## Biographical Information

*Yujie Zhu obtained her Ph.D. degree in Chemistry from Nankai University in 2018 under the supervision of Professor Zhijin Fan. In 2018, she joined the team of Professor Julius Rebek, Jr. and Professor Yang Yu at Shanghai University, focusing her research on the synthesis of new water‐soluble funcionalized deep cavitands, moleculer recognition and reactivity in confined cavity. Since 2021, she joined the faculty of the department of chemistry at Shanghai University*.

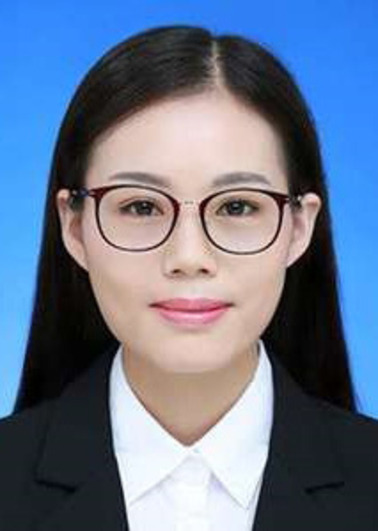

## Biographical Information

*Mingkai Zhao received his BS degree in chemistry from Hunan University in 2018. Currently, he is a master student in the group of Professor Julius Rebek Jr. and Yang Yu at Shanghai University. His research interests are supramolecular chemistry based on organic synthesis*.

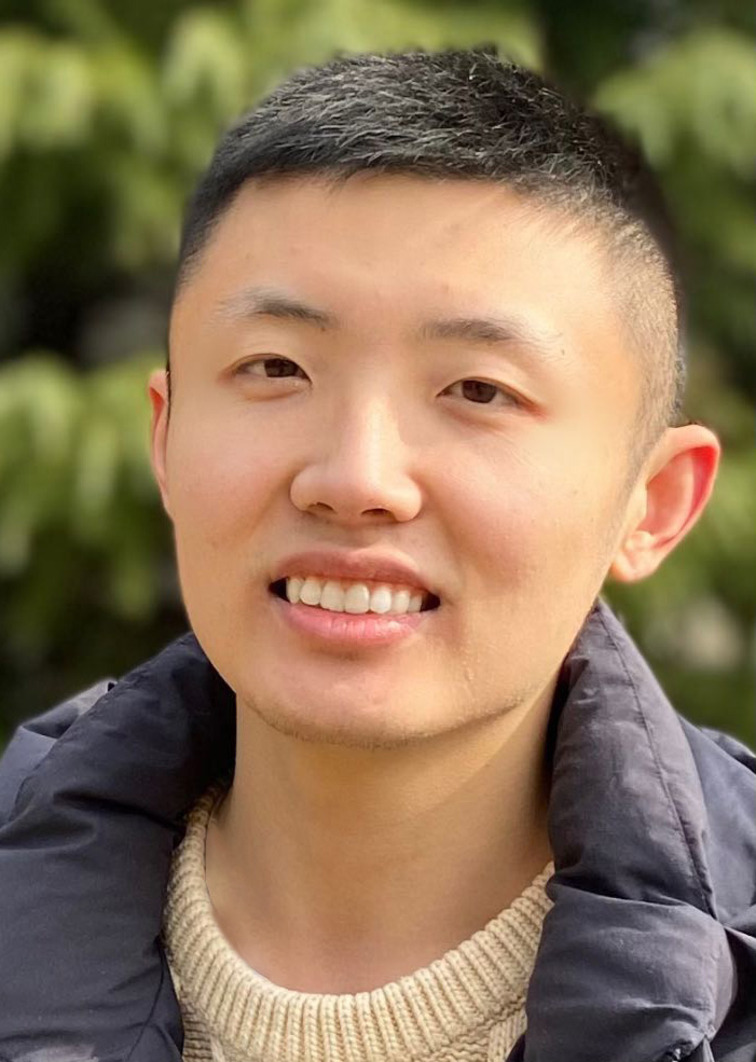

## Biographical Information

*Yang Yu received her Ph.D. in 2013 from Heidelberg University working under the direction of Professor Stephen Hashmi and then moved to UT Southwestern Medical Center as a post‐doctoral researcher with Professor Uttam Tambar. In 2015, she joined the group of Professor Julius Rebek, Jr., at The Scripps Research Institute as a research associate then started her independent career as a professor in Shanghai University in 2017. Her current research interests lie in molecular recognition and catalysis*.

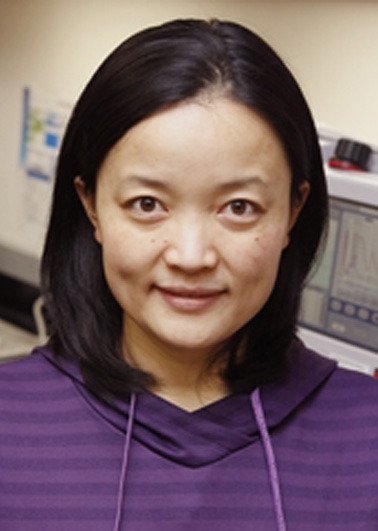

## Biographical Information

*Julius Rebek, Jr., is the Director Emeritus of the Skaggs Institute for Chemical Biology and at the Scripps Research Institute and special professor at Shanghai University since 2021. He received his Ph.D. degree in chemistry from MIT and has held professorships at UCLA, the University of Pittsburgh, and MIT. He is a member of the U.S. National Academy of Sciences, and honorary member of the Hungarian Academy of Sciences and the Royal Swedish Academy. His current research interests include self‐assembling systems and molecular behavior in small spaces*.

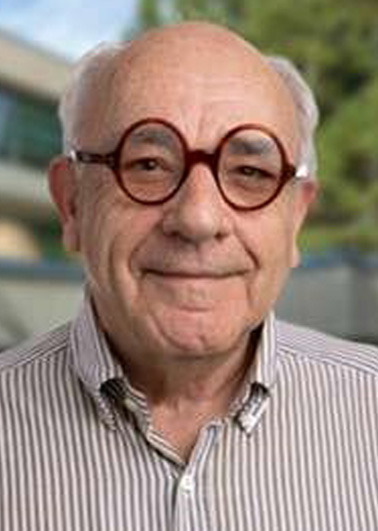

## Data Availability

Data sharing is not applicable to this article as no new data were created or analyzed in this study.

## References

[1] B. Baruahab , P. Deb , Mater. Adv. 2021, 2, 5344–5366.

[2] P. Ball, *H_2_O: A Biography of Water*; Weidenfeld & Nicolson, London, UK.

[3] G. V. Oshovsky , D. N. Reinhoudt , W. Verboom , Angew. Chem. Int. Ed. 2007, 46, 2366–2393; 10.1002/anie.200602815 17370285

[4a] D. Fiedler , D. H. Leung , R. G. Bergman , K. N. Raymond , Acc. Chem. Res. 2005, 38, 349–358; 15835881 10.1021/ar040152p

[4b] M. Fujita , M. Tominaga , A. Hori , B. Therrien , Acc. Chem. Res. 2005, 38, 371–380; 10.1021/ar040153h 15835883

[4c] S. Liu , B. C. Gibb , Chem. Commun. 2008, 3709–3716; 10.1039/b805446k PMC2614893 18685753

[4d] M. M. Conn , J. Rebek , Chem. Rev. 1997, 97, 1647–1668; 11851461 10.1021/cr9603800

[4e] F. Hof , S. L. Craig , C. Nuckolls , J. Rebek , Angew. Chem. Int. Ed. 2002, 41, 1488–1508; 10.1002/1521-3773(20020503)41:9<1488::aid-anie1488>3.0.co;2-g 19750648

[4f] J. R. Nitschke , Acc. Chem. Res. 2007, 40, 103–112. 17309191 10.1021/ar068185n

[5a] L. Adriaenssens , P. Ballester , Chem. Soc. Rev. 2013, 42, 3261–3277; 23321897 10.1039/c2cs35461f

[5b] L. Avram , Y. Cohen , J. Rebek , Chem. Commun. 2011, 47, 5368–5375; 10.1039/c1cc10150a 21431233

[5c] Q. Zhang , L. Catti , K. Tiefenbacher , Acc. Chem. Res. 2018, 51, 2107–2114. 30153000 10.1021/acs.accounts.8b00320

[6a] J. R. Moran , S. Karbach , D. J. Cram , J. Am. Chem. Soc. 1982, 104, 5826–5828;

[6b] P. Soncini , S. Bonsignore , E. Dalcanale , F. Ugozzoli , J. Chem. Soc. Chem. Commun. 1989, 1989, 500–502;

[6c] H. Erdtman , S. Höberg , S. Abrahamsson , B. Nilsson , Tetrahedron Lett. 1968, 9, 1679–1682;

[6d] D. M. Rudkevich , G. Hilmersson , J. Rebek , J. Am. Chem. Soc. 1997, 119, 9911–9912;

[6e] C. L. D. Gibb , E. D. Stevens , B. C. Gibb , J. Am. Chem. Soc. 2001, 123, 5849–5850. 11403638 10.1021/ja005931p

[7a] S. Chen , M. Yamasaki , S. Polen , J. Gallucci , C. M. Hadad , J. D. Badjìc , J. Am. Chem. Soc. 2015, 137, 12276–12281; 26348904 10.1021/jacs.5b06041

[7b] C. L. D. Gibb , B. C. Gibb , J. Am. Chem. Soc. 2004, 126, 11408–11409; 15366865 10.1021/ja0475611

[7c] L. Trembleau , J. Rebek , Science 2003, 301, 1219–1220; 12947192 10.1126/science.1086644

[7d] M. D. Giles , S. Liu , R. L. Emanuel , B. C. Gibb , S. M. Grayson , J. Am. Chem. Soc. 2008, 130, 14430–14431; 18847205 10.1021/ja806457x PMC2744078

[7e] A. Lledo , J. Rebek , Chem. Commun. 2010, 46, 8630–8632; 10.1039/c0cc03388j 20941420

[7f] K. D. Zhang , D. Ajami , J. V. Gavette , J. Rebek , J. Am. Chem. Soc. 2014, 136, 5264–5266. 24666031 10.1021/ja501685z

[8a] Y. Yu , J. Rebek , Acc. Chem. Res. 2018, 51, 3031–3040; 30398326 10.1021/acs.accounts.8b00269

[8b] Y. Yu , J. M. Yang , J. Rebek , Chem. 2020, 6, 1265–1274.

[9] K. D. Zhang , D. Ajami , J. Rebek , J. Am. Chem. Soc. 2013, 135, 18064–18066. 24245649 10.1021/ja410644p

[10a] K. D. Zhang , D. Ajami , J. V. Gavette , J. Rebek , Chem. Commun. 2014, 50, 4895–4897; 10.1039/c4cc01643b 24687171

[10b] J. Rebek , Y. Yu , S. Mosca , Heterocycles. 2017, 95, 127–130.

[11a] S. Mosca , Y. Yu , J. V. Gavette , J. Rebek , J. Am. Chem. Soc. 2015, 137, 14582–14585; 26540097 10.1021/jacs.5b10028

[11b] Q. Shi , D. Masseroni , J. Rebek , J. Am. Chem. Soc. 2016, 138, 10846–10848; 27529442 10.1021/jacs.6b06950

[11c] N. W. Wu , I. D. Petsalakis , G. Theodorakopoulos , Y. Yu , J. Rebek , Angew. Chem. Int. Ed. 2018, 57, 15091–15095; 10.1002/anie.201808265 30246478

[12a] A. Radzicka , L. Pedersen , R. Wolfenden , Biochemistry 1988, 27, 4538–4541; 3166998 10.1021/bi00412a047

[12b] W. L. Jorgensen , J. Gao , J. Am. Chem. Soc. 1988, 110, 4212–4216;

[12c] L. A. LaPlanche , M. T. Rogers , J. Am. Chem. Soc. 1964, 86, 337–341.

[13] Y. S. Li , L. Escobar , Y. J. Zhu , Y. Cohen , P. Ballester , J. Rebek , Y. Yu , Proc. Natl. Acad. Sci. USA 2019, 116, 19815–19820. 31527234 10.1073/pnas.1911331116 PMC6778222

[14] L. Escobar , Y. S. Li , Y. Cohen , Y. Yu , J. Rebek , P. Ballester , Chem. Eur. J. 2020, 26, 8220–8225. 32167599 10.1002/chem.202000781

[15a] D. A. Dougherty , Acc. Chem. Res. 2013, 46, 885–893; 23214924 10.1021/ar300265y PMC3957424

[15b] J. C. Ma , D. A. Dougherty , Chem. Rev. 1997, 97, 1303–1324. 11851453 10.1021/cr9603744

[16a] J. H. Jordan , B. C. Gibb , Chem. Soc. Rev. 2015, 44, 547–85; 25088697 10.1039/c4cs00191e

[16b] J. Murray , K. Kim , T. Ogoshi , W. Yao , B. C. Gibb , Chem. Soc. Rev. 2017, 46, 2479–2496. 28338130 10.1039/c7cs00095b PMC5462124

[17a] K. S. Kim , J. Y. Lee , S. J. Lee , T.-K. Ha , D. H. Kim , J. Am. Chem. Soc. 1994, 116, 7399–7400;

[17b] C. A. Deakyne , M. Meot-Ner , J. Am. Chem. Soc. 1985, 107, 474–479;

[17c] S. Mecozzi , P. West , J. D. A. Dougherty , J. Am. Chem. Soc. 1996, 118, 2307–2308.

[18a] S. M. Biros , E. C. Ullrich , F. Hof , L. Trembleau , J. Rebek , J. Am. Chem. Soc. 2004, 126, 2870–2876; 14995204 10.1021/ja038823m

[18b] A. Lascaux , G. Leener , L. Fusaro , F. Topic , K. Rissanen , M. Luhmer , I. Jabin , Org. Biomol. Chem. 2016, 14, 738–746; 26580493 10.1039/c5ob02067k

[18c] G. Penuelas-Haro , P. Ballester , Chem. Sci. 2019, 10, 2413–2423; 30931096 10.1039/c8sc05034a PMC6399678

[18d] G. B. Huang , S. H. Wang , H. Ke , L. P. Yang , W. Jiang , J. Am. Chem. Soc. 2016, 138, 14550–14553; 27792319 10.1021/jacs.6b09472

[18e] C. Ke , H. Destecroix , M. P. Crump , A. P. A. Davis , Nat. Chem. 2012, 4, 718–723. 22914192 10.1038/nchem.1409

[19] Y. J. Zhu , M. M. Tang , H. B. Zhang , F. Rahman , P. Ballester , J. Rebek , C. A. Hunter , Y. Yu , J. Am. Chem. Soc. 2021, 143, 12397–12403. 34328320 10.1021/jacs.1c06510

[20a] J. Scheirs, T. E. Long, *Industrial Modern Polyesters: Chemistry and Technology of Polyesters and Copolyesters*; Wiley, Chichester, **2003**;

[20b] D. S. Sholl , R. P. Lively , Nature. 2016, 532, 435–437. 27121824 10.1038/532435a

[21a] K. Chen , T. Sun , H. Xiang , Can. J. Chem. Eng. 2016, **94**, 128–133;

[21b] J. Liu , S. Zhao , X. Chen , B. Shen , Fuel 2016, 166, 467–472.

[22] G. C. Laredo , J. L. Cano , J. Castillo , J. A. Hernandez , J. O. Marroquin , Fuel 2014, 117, 660–666.

[23a] D. Dubbeldam , C. J. Galvin , K. S. Walton , D. E. Ellis , R. Q. Snurr , J. Am. Chem. Soc. 2008, 130, 10884–10885; 18651737 10.1021/ja804039c

[23b] Z. R. Herm , B. M. Wiers , J. A. Mason , J. M. van Baten , M. R. Hudson , P. Zajdel , C. M. Brown , N. Masciocchi , R. Krishna , J. R. Long , Science 2013, 340, 960–964. 23704568 10.1126/science.1234071

[24a] T. Ogoshi , K. Saito , R. Sueto , R. Kojima , Y. Hamada , S. Akine , A. M. P. Moeljadi , H. Hirao , T. Kakuta , T. A. Yamagishi , Angew. Chem. Int. Ed. 2018, 57, 1592–1595; 10.1002/anie.201711575 29251391

[24b] K. Jie , M. Liu , Y. Zhou , M. A. Little , A. Pulido , S. Y. Chong , A. Stephenson , A. R. Hughes , F. Sakakibara , T. Ogoshi , F. Blanc , G. M. Day , F. Huang , A. I. Cooper , J. Am. Chem. Soc. 2018, 140, 6921–6930. 29754488 10.1021/jacs.8b02621 PMC5997404

[25] F.-U. Rahman , H.-N. Feng , Y. Yu , Org. Chem. Front. 2019, 6, 998–1001.

[26] F. U. Rahman , Y. S. Li , I. D. Petsalakis , G. Theodorakopoulos , J. Rebek , Y. Yu , Proc. Natl. Acad. Sci. USA 2019, 116, 17648–17653. 31427538 10.1073/pnas.1909154116 PMC6731629

[27] S. Korom , P. Ballester , J. Am. Chem. Soc. 2017, 139, 12109–12112. 28820939 10.1021/jacs.7b05458

[28a] P. Roncucci , L. Pirondini , G. Paderni , C. Massera , E. Dalcanale , V. A. Azov , F. Diederich , Chem. Eur. J. 2006, 12, 4775–4784; 16671048 10.1002/chem.200600085

[28b] T. Iwasawa , Tetrahedron Lett. 2017, 58, 4217–4226.

[29] F. U. Rahman , J.-M. Yang , Y.-H. Wan , H.-B. Zhang , I. D. Petsalakis , G. Theodorakopoulos , J. Rebek , Y. Yu , Chem. Commun. 2020, 56, 6945–6948. 10.1039/d0cc02778b 32436496

[30] G. Zhang , A.-H. Emwas , U. F. S. Hameed , S. T. Arold , P. Yang , A. Chen , J.-F. Xiang , N. M. Khashab , Chem. 2020, 6, 1082–1096.

[31] Y.-H. Wan , F.-U. Rahman , J. Rebek , Y. Yu Chin , J. Chem. 2021, 39, 1498–1502.

[32] A. Wishard , B. C. Gibb in: Calixarenes and Beyond (Eds. P. Neri , J. L. Sessler , M.-X. Wang ), Springer, 2016, 195–234.

[33] H.-B. Zhang , K. Kanagaraj , J. Rebek , Y. Yu , J. Org. Chem. 2021, 86, 8873–8881. 34114823 10.1021/acs.joc.1c00794

[34] K. Wang , X. Cai , W. Yao , D. Tang , R. Kataria , H. S. Ashbaugh , L. D. Byers , B. C. Gibb , J. Am. Chem. Soc. 2019, 141, 6740–6747. 30929421 10.1021/jacs.9b02287

[35] J.-M. Yang , Y.-Q. Chen , Y. Yu , P. Ballester , J. Rebek , J. Am. Chem. Soc. 2021, 143, 19517–19524. 34762414 10.1021/jacs.1c09226

[36a] D. Loomis , K. Guyton , Y. Grosse , F. El Ghissasi , V. Bouvard , L. Benbrahim-Tallaa , N. Guha , H. Mattock , K. Straif , Lancet Oncol. 2015, 16, 891–892; 27784619 10.1016/S1470-2045(16)30513-7

[36b] H. Parada , M. S. Wolﬀ , L. S. Engel , A. J. White , S. M. Eng , R. Cleveland , N. K. Khankari , S. L. Teitelbaum , A. I. Neugut , M. D. Gammon , Int. J. Cancer 2016, 138, 565–575; 26285160 10.1002/ijc.29806 PMC4715584

[36c] N. Roswall , M. Sorensen , A. Tjonneland , O. Raaschou-Nielsen , Environ. Res. 2018, 163, 237–248. 29459306 10.1016/j.envres.2018.02.003

[37] H. N. Feng , M. Petroselli , X.-H. Zhang , J. Rebek , Y. Yu , Supramol. Chem. 2019, 31, 108–113.

[38] I. D. Petsalakis , D. Tzeli , G. Theodorakopoulos , J. Rebek , Chem. Phys. Lett. 2019, 728, 174–180.

[39a] R. J. Hooley , J. V. Gavette , M. Mettry , D. Ajami , J. Rebek , Chem. Sci. 2014, 5, 4382–4387;

[39b] Y. Yu , Y.-S. Li , J. Rebek , New J. Chem. 2018, 42, 9945–9948;

[39c] N. Endo , M. Inoue , T. Iwasawa , Eur. J. Org. Chem. 2018, 2018, 1136–1140.

[40a] H. J. Schneider , A. K. Yatsimirsky , Chem. Soc. Rev. 2008, 37, 263–277; 18197343 10.1039/b612543n

[40b] H. H. L. Lee , J. W. Lee , Y. Jang , Y. H. Ko , K. Kim , H. I. Kim , Angew. Chem. Int. Ed. 2016, 55, 8249–8253; 10.1002/anie.201601320 27192972

[41a] Y. Ferrand , M. P. Crump , A. P. Davis , Science 2007, 318, 619–622; 17962557 10.1126/science.1148735

[41b] B. Verdejo , G. Gil-Ramírez , P. Ballester , J. Am. Chem. Soc. 2009, 131, 3178–3179; 19216565 10.1021/ja900151u

[41c] C. Ke , H. Crump , M. P. Destecroix , A. P. Davis , Nat. Chem. 2012, 4, 718–723; 22914192 10.1038/nchem.1409

[41d] E. M. Peck , W. Liu , G. T. Spence , S. K. Shaw , A. P. Davis , H. Destecroix , B. D. Smith , J. Am. Chem. Soc. 2015, 137, 8668–8671; 26106948 10.1021/jacs.5b03573 PMC4643739

[41e] T. J. Mooibroek , J. M. Casas-Solvas , R. L. Harniman , C. M. Renney , T. S. Carter , M. P. Crump , A. P. Davis , Nat. Chem. 2016, 8, 69–74. 26673266 10.1038/nchem.2395 PMC5856347

[42] H.-W. Guan , Y.-J. Zhu , F. Himo , J. Rebek , Y. Yu , Chem. Commun. 2021, 57, 8147–8150. 10.1039/d1cc02505h 34312642

[43] Y.-H. Wan , Y. J. Zhu , J. Rebek , Y. Yu , Molecules 2021, 26, 1922. 33808102 10.3390/molecules26071922 PMC8037811

[44] M. Petroselli , Y.-Q. Chen , J. Rebek , Y. Yu , Green Synth. Catal. 2021, 2, 123–130.

[45] Q. Shi , M. P. Mower , D. G. Blackmond , J. Rebek , Proc. Natl. Acad. Sci. USA 2016, 113, 9199–9203. 27482089 10.1073/pnas.1610006113 PMC4995975

[46] N. W. Wu , J. Rebek , J. Am. Chem. Soc. 2016, 138, 7512–7515. 27259017 10.1021/jacs.6b04278

[47] D. Masseroni , S. Mosca , M. P. Mower , D. G. Blackmond , J. Rebek , Angew. Chem. Int. Ed. 2016, 55, 8290–8293; 10.1002/anie.201602355 27254667

[48] V. Angamuthu , M. Petroselli , F. U. Rahman , Y. Yu , J. Rebek , Org. Biomol. Chem. 2019, 17, 5279–5282. 31090780 10.1039/c9ob01018a

[49] V. Angamuthu , F. U. Rahman , M. Petroselli , Y. S. Li , Y. Yu , J. Rebek , Org. Chem. Front. 2019, 6, 3220–3223.

[50] L. S. Kaanumalle , C. L. Gibb , B. C. Gibb , V. Ramamurthy , J. Am. Chem. Soc. 2004, 126, 14366–14367. 15521751 10.1021/ja0450197

[51] A. K. Sundaresan , C. L. Gibb , B. C. Gibb , V. Ramamurthy , Tetrahedron 2009, 65, 7277–7288. 21037975 10.1016/j.tet.2009.01.110 PMC2964841

[52] V. Ramamurthy , Acc. Chem. Res. 2015, 48, 2904–2917. 26488308 10.1021/acs.accounts.5b00360

[53] M. Petroselli , V. Angamuthu , F.-U. Rahman , Y. Yu , J. Rebek , J. Am. Chem. Soc. 2020, 142, 2396–2403. 31913618 10.1021/jacs.9b11595

[54] M. Petroselli , Y. Yu , J. Rebek , Chem. Eur. J. 2021, 27, 3284–3287. 33301606 10.1002/chem.202004953

[55] H. Takezawa , T. Kanda , H. Nanjo , M. Fujita , J. Am. Chem. Soc. 2019, 141, 5112–5115. 30874439 10.1021/jacs.9b00131

[56] M. Morimoto , S. M. Bierschenk , K. T. Xia , R. G. Bergman , K. N. Raymond , D. Toste , Nat. Catal. 2020, 3, 969–984.

[57] Q. Q. Sun , L. Escobar , P. Ballester , Angew. Chem. Int. Ed. 2021, 60, 10359–10365. 10.1002/anie.202101499 33596326

[58] Y. Yu , J. M. Yang , J. Rebek , Chem. 2020, 6, 1265–1274.

[59] Y. Yu , J. Rebek , Acc. Chem. Res. 2018, 51, 3031–3040. 30398326 10.1021/acs.accounts.8b00269

[60] J. M. Yang , Y. Yu , J. Rebek , J. Am. Chem. Soc. 2021, 143, 2190–2193. 33507732 10.1021/jacs.0c12302

